# Enhanced decolourization of the azo dye Sirius rose BB by laccase–HBT system

**DOI:** 10.1007/s13205-011-0041-5

**Published:** 2011-12-16

**Authors:** Ouafa Benzina, Fakher Frikha, Héla Zouari-Mechichi, Steve Woodward, Lassaad Belbahri, Emna Mnif, Tahar Mechichi

**Affiliations:** 1 Ecole Nationale d’Ingénieurs de Sfax, Université de Sfax, Route de Soukra Km 4,5 BP 1173, 3038 Sfax, Tunisia; 2 Department of Plant and Soil Science, Institute of Biological and Environmental Sciences, University of Aberdeen, Cruickshank Building, St. Machar Drive, Aberdeen, AB24 3UU Scotland, UK; 3 Laboratory of Soil Biology, University of Neuchatel, Rue Emile Argand 11, 2009 Neuchatel, Switzerland

**Keywords:** Laccase, Dyes, Box–Behnken, Decolourization, Response surface

## Abstract

Response surface methodology (RSM) was applied to optimize the decolourization of the diazo dye Sirius rose BB (SR) by crude laccase from the white-rot fungus *Trametes* sp. strain CLBE55. A Box–Behnken design using RSM with six variables, namely pH, incubation temperature, enzyme (laccase) concentration, 1-hydroxybenzotriazol (HBT) concentration, dye concentration and incubation time was used in this study to determine significant correlations between the effects of these variables on the decolourization of Sirius rose. The optimum concentrations of HBT, dye and laccase were 0.5 mM, 60 mg/L and 0.1 U/mL, respectively, to obtain maximum decolourization of Sirius rose (approx. 99.5% in 150 min at 45 °C, pH 3). A quadratic model was obtained for dye decolourization using this design. Experimental values were in good agreement with values predicted by the model, giving highly significant correlations.

## Introduction

The treatment of textile effluents is of paramount importance due to toxic and aesthetic impacts on receiving waters. While much research has been focused on the development of effective treatment technologies for wastewaters containing azo dyes, no single solution has been effective in the remediating the broad diversity of textile wastes. Human and ecological health concerns have prompted governments to require textile effluent discharges to have increasingly lower colour and nitrogen concentrations. Despite being aware of this problem, many textile manufacturers have failed to adequately remove azo dye compounds from their wastewaters. Until dye and textile manufactures are able to develop efficient technologies, allowing for increased dye–fiber bonding and lower dyehouse losses (Lewis et al. 1999), the problem of treating these types of wastes will fall to the wastewater treatment facilities.

Azo dyes are synthetic organic compounds widely used in textile dyeing. This chemical class of dyes, which is characterized by the presence of at least one azo bond (N=N) bearing aromatic rings, dominates the world market in dyestuffs, with a share of approximately 70% (Soares et al. 2002). These dyes have high photolytic stability and resistance towards major oxidising agents (Reife and Othmer 1993).

The release of azo dyes into the environment in effluent from textile dyeing plants is a major concern in wastewater treatment, since these compounds are highly recalcitrant to conventional wastewater treatment processes. The recalcitrance of azo dyes has been attributed to the presence of sulfonate groups and azo bonds, two features generally considered as xenobiotic (Rieger et al. 2002). In addition, some azo dyes or their metabolites may be mutagens or carcinogens (McCann and Ames 1976). Several combined anaerobic and aerobic microbial treatments have been suggested to enhance the degradation of azo dyes (Neill et al. 2002). Under anaerobic conditions, however, azo reductases usually cleave azo dyes into the corresponding amines, many of which are also mutagenic and/or carcinogenic (Chung and Cerniglia 1992; Abadulla et al. 2000). Furthermore, azo-reductases have been shown to be highly specific enzymes, cleaving only azo bonds of selected dyes (Zimmermann et al. 1984).

Altogether these problems underline the need for nonspecific processes for the effective treatment of wastewater containing these types of dyes.

Laccases (*p*-diphenol: dioxygen oxidoreductases; EC 1.10.3.2) are copper-containing enzymes that catalyze the one electron oxidation of phenolic substrates and aromatic amines.

It has also been shown that, in the presence of appropriate low-molecular-weight compounds (redox mediators), laccases are able to oxidize a wide range of other aromatic compounds (Bourbonnais and Paice 1990) therefore expanding the range of compounds that can be oxidized by these enzymes. Laccases are particularly abundant in white-rot fungi, which are the living organisms most effective in the degradation of whole wood components (Kirk et al. 1982). In particular, the genus *Trametes* appears to include some of the most efficient lignin degraders.

A number of statistically designed experimental models have been applied to optimize culture parameters in biological research. RSM, first described by Box and Wilson (Box et al. 1978), is an experimental approach used to identify the optimum conditions for a multivariable system. This methodology has been successfully applied in the optimization of the culture parameters in ligninolytic enzyme production and dye decolourization with fungal cultures (Giovanni 1983; Carlson 1992). Recently, RSM has been applied for the optimization of redox mediator concentrations in laccase-mediated pulp bleaching (Cristóvão et al. 2008). In enzymatic dye decolourization, optimization of the concentrations of redox mediator, dye and enzyme is an important criterion for successful decolourization.

In this paper, RSM was applied to optimize the decolourization of the diazo dye Sirius rose BB (SR) by crude laccase from the white-rot fungus *Trametes* sp. strain CLBE55. Box–Behnken design using RSM with six variables (pH, temperature, incubation time, laccase concentration, HBT concentration and dye concentration) was used to optimize significant correlations between the effects of these variables on the decolourization of SR.

## Materials and methods

### Chemicals

**Fig. 1:**
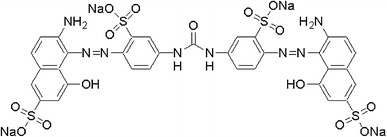
Chemical structure of the diazo dye Sirius rose BB (Chlorazol fast pink)

2,6-Dimethoxyphenol (DMP), 1-hydroxybenzotriazole (HBT) and Dye Sirius Rose BB (Chlorazol fast pink) (Fig. 1), colour index number: 25388, were obtained from Sigma-Aldrich.

### Fungal strain, media and culture conditions

The fungal strain used in this study was *Trametes* sp. strain CLBE55 (Maalej-Kammoun et al. 2009). Fungal isolates are maintained in the culture collection of our laboratory. For short term conservation, isolates were maintained on 2% malt extract, 1% agar in Petri dishes, cultured at 30 °C and stored at 4 °C.

Laccase production by *Trametes* sp. strain CLBE55 was performed in basal liquid medium (Munoz et al. 1997), containing (per litre): glucose, 10 g; peptone, 5 g; yeast extract, 1 g; ammonium tartrate, 2 g; KH_2_PO_4_, 1 g; MgSO_4_. 7H_2_O, 0.5 g; KCl, 0.5 g; trace element solution, 1 mL. The trace element solution comprises (per litre): B_4_O_7_Na_2_·10H_2_O, 0.1 g; CuSO_4_·5H_2_O, 0.01 g; FeSO_4_·7H_2_O, 0.05 g; MnSO_4_·7H_2_O; 0.01 g; ZnSO_4_·7H_2_O, 0.07 g; (NH_4_)_6_Mo_7_O_24_·4H_2_O, 0.01 g. The pH of the basal medium was adjusted to 5.5 before dispensing in 300 mL volumes into 1 L Erlenmeyer flasks. After autoclaving at 105 kPa for 20 min, 3 mL of homogenized mycelium were used for inoculation of the flasks. CuSO_4_ (300 μM) was added to the basal medium to stimulate the production of laccase. Cultures were incubated at 30 °C on a rotary shaker (160 rpm).

### Enzyme assay

After centrifuging the medium, laccase activity was assayed using 10 mM 2,6-dimethoxyphenol (DMP) in 100 mM acetate buffer, pH 5 (ε_469 nm_ = 27,500 M cm^−1^, referenced to DMP concentration) (Munoz et al. 1997). The reactions were carried out at room temperature (22–25 °C). One unit of laccase activity was defined as the amount of enzyme oxidi*z*ing 1 μmol of substrate per minute.

### Dye decolourization experiments

All experiments were performed using 50 mL-disposable flasks in 5 mL final reaction volume. The reaction mixture contained 100 mM citrate buffer, HBT, dye and laccase from culture filtrates. The reaction was initiated by the addition of laccase and incubated in the dark. Decolourization of SR was followed by measuring absorbance at the wave length of maximum dye absorbance (600 nm) at 30 min intervals. All experiments were performed in duplicate; controls did not contain laccase.

pH of the citrate buffer, temperature, laccase concentration, dye concentration, HBT concentration and incubation time were the parameters optimized (see below).where Absorbance_*t*0_ is the absorbance of the reaction mixture at the wavelength of maximum absorbance by the dye before incubation with the enzyme and Absorbance_*t*f_ is the absorbance of the reaction mixture after incubation.

### Experimental designs: Box–Behnken design

Experimental conditions for optimization of the decolourization yield of the dye Sirius rose were determined using RSM. Six selected experimental variables (pH, Temperature (°C), enzyme concentration (U/mL), HBT concentration (mM), dye concentration (mg/L) and incubation time (min)) were chosen and assessed at three coded levels (−1, 0 and +1), as shown in Table 1.

**Table 1:** Range of variables for the experimental design

Variable		Level		
		−1	0	1
*X*_1_	pH	3	4.5	6
*X*_2_	Temperature (°C)	30	45	60
*X*_3_	Enzyme concentration (U/mL)	0.1	0.5	0.9
*X*_4_	HBT concentration (mM)	0.1	0.5	0.9
*X*_5_	Dye concentration (mg/L)	20	60	100
*X*_6_	Incubation time (min)	30	90	150

To define the optimum settings for these factors, a Box–Behnken design was applied in the experiments (Table 2). The response was measured in terms of decolourization yield (Table 3). This response can be described by the following second order model (Eq. 1) adequate for predicting the response in the experimental region:1with *y* =  + *e*, where *e* represents deviation between measured response (*y*) and estimated response (); *b*_0_, *b*_*i*_, *b*_*ij*_, and *b*_*ii*_ are estimated model coefficients; and *X*_i_ represents coded variables.

**Table 2:** Six-variable Box–Behnken experimental design

Run	*X*_1_	*X*_2_	*X*_3_	*X*_4_	*X*_5_	*X*_6_	Run	*X*_1_	*X*_2_	*X*_3_	*X*_4_	*X*_5_	*X*_6_
1	−1	−1	0	−1	0	0	28	1	0	0	1	−1	0
2	1	−1	0	−1	0	0	29	−1	0	0	−1	1	0
3	−1	1	0	−1	0	0	30	1	0	0	−1	1	0
4	1	1	0	−1	0	0	31	−1	0	0	1	1	0
5	−1	−1	0	1	0	0	32	1	0	0	1	1	0
6	1	−1	0	1	0	0	33	0	−1	0	0	−1	−1
7	−1	1	0	1	0	0	34	0	1	0	0	−1	−1
8	1	1	0	1	0	0	35	0	−1	0	0	1	−1
9	0	−1	−1	0	−1	0	36	0	1	0	0	1	−1
10	0	1	−1	0	−1	0	37	0	−1	0	0	−1	1
11	0	−1	1	0	−1	0	38	0	1	0	0	−1	1
12	0	1	1	0	−1	0	39	0	−1	0	0	1	1
13	0	−1	−1	0	1	0	40	0	1	0	0	1	1
14	0	1	−1	0	1	0	41	−1	0	−1	0	0	−1
15	0	−1	1	0	1	0	42	1	0	−1	0	0	−1
16	0	1	1	0	1	0	43	−1	0	1	0	0	−1
17	0	0	−1	−1	0	−1	44	1	0	1	0	0	−1
18	0	0	1	−1	0	−1	45	−1	0	−1	0	0	1
19	0	0	−1	1	0	−1	46	1	0	−1	0	0	1
20	0	0	1	1	0	−1	47	−1	0	1	0	0	1
21	0	0	−1	−1	0	1	48	1	0	1	0	0	1
22	0	0	1	−1	0	1	49	0	0	0	0	0	0
23	0	0	−1	1	0	1	50	0	0	0	0	0	0
24	0	0	1	1	0	1	51	0	0	0	0	0	0
25	−1	0	0	−1	−1	0	52	0	0	0	0	0	0
26	1	0	0	−1	−1	0	53	0	0	0	0	0	0
27	−1	0	0	1	−1	0	54	0	0	0	0	0	0

*X*_1_ pH, *X*_2_ temperature, *X*_3_ enzyme concentration, *X*_4_ HBT concentration, *X*_5_ dye concentration and *X*_6_ incubation time

**Table 3:** Experimental conditions of the Box–Behnken design and the corresponding experimental responses

Run	*X*_1_	*X*_2_	*X*_3_	*X*_4_	*X*_5_	*X*_6_	*Y*	Run	*X*_1_	*X*_2_	*X*_3_	*X*_4_	*X*_5_	*X*_6_	*Y*
1	3.0	30	0.5	0.1	60	90	84.21	28	6.0	45	0.5	0.9	20	90	89.53
2	6.0	30	0.5	0.1	60	90	26.92	29	3.0	45	0.5	0.1	100	90	94.21
3	3.0	60	0.5	0.1	60	90	80.24	30	6.0	45	0.5	0.1	100	90	72.78
4	6.0	60	0.5	0.1	60	90	77.72	31	3.0	45	0.5	0.9	100	90	84.90
5	3.0	30	0.5	0.9	60	90	87.73	32	6.0	45	0.5	0.9	100	90	92.01
6	6.0	30	0.5	0.9	60	90	69.95	33	4.5	30	0.5	0.5	20	30	84.26
7	3.0	60	0.5	0.9	60	90	69.78	34	4.5	60	0.5	0.5	20	30	89.53
8	6.0	60	0.5	0.9	60	90	90.65	35	4.5	30	0.5	0.5	100	30	89.91
9	4.5	30	0.1	0.5	20	90	89.95	36	4.5	60	0.5	0.5	100	30	92.81
10	4.5	60	0.1	0.5	20	90	86.04	37	4.5	30	0.5	0.5	20	150	86.04
11	4.5	30	0.9	0.5	20	90	80.21	38	4.5	60	0.5	0.5	20	150	64.36
12	4.5	60	0.9	0.5	20	90	75.20	39	4.5	30	0.5	0.5	100	150	91.68
13	4.5	30	0.1	0.5	100	90	91.66	40	4.5	60	0.5	0.5	100	150	90.05
14	4.5	60	0.1	0.5	100	90	95.07	41	3.0	45	0.1	0.5	60	30	90.26
15	4.5	30	0.9	0.5	100	90	89.80	42	6.0	45	0.1	0.5	60	30	16.00
16	4.5	60	0.9	0.5	100	90	87.00	43	3.0	45	0.9	0.5	60	30	89.40
17	4.5	45	0.1	0.1	60	30	91.62	44	6.0	45	0.9	0.5	60	30	68.03
18	4.5	45	0.9	0.1	60	30	91.59	45	3.0	45	0.1	0.5	60	150	96.34
19	4.5	45	0.1	0.9	60	30	88.70	46	6.0	45	0.1	0.5	60	150	85.29
20	4.5	45	0.9	0.9	60	30	85.04	47	3.0	45	0.9	0.5	60	150	81.32
21	4.5	45	0.1	0.1	60	150	99.51	48	6.0	45	0.9	0.5	60	150	92.52
22	4.5	45	0.9	0.1	60	150	98.67	49	4.5	45	0.5	0.5	60	90	88.78
23	4.5	45	0.1	0.9	60	150	92.59	50	4.5	45	0.5	0.5	60	90	89.18
24	4.5	45	0.9	0.9	60	150	80.44	51	4.5	45	0.5	0.5	60	90	88.63
25	3.0	45	0.5	0.1	20	90	67.39	52	4.5	45	0.5	0.5	60	90	89.63
26	6.0	45	0.5	0.1	20	90	79.06	53	4.5	45	0.5	0.5	60	90	88.78
27	3.0	45	0.5	0.9	20	90	76.00	54	4.5	45	0.5	0.5	60	90	88.58

*X*_1_ pH, *X*_2_ temperature (°C), *X*_3_ enzyme concentration (U/mL), *X*_4_ HBT concentration (mM), *X*_5_ dye concentration (mg/L) and *X*_6_ incubation time (min), *Y* Sirius rose BB yield of decolourization (%)

To estimate the model coefficients, a six-variable Box–Behnken design, requiring 54 experiments (Table 2), was carried out in a cubic experimental domain. The experimental points are located in the middle of the cube ridges (48 experiments) and in the center of the cube (6 experiments) (Myers and Montgomery 1995; Goupy 1999). The adequacy of the model was tested using seven check points. The fitted model was used to estimate the relative sensitivity of the response to the variables and to examine the optimal experimental conditions. The relationships between the responses and the experimental variables were illustrated graphically by plotting the response surfaces and the isoresponse curves (Myers and Montgomery 1995). In this study, the generation and the data treatment of the Box–Behnken design were performed using the experimental design software Design-Expert^®^ Version 7.0.0.

## Results and discussion

### Experimental design data

To carry out the Box–Behnken experimental design, high and low levels were chosen for each factor (Table 2). Table 3 shows the actual experimental conditions used in the Box–Behnken design with the corresponding measured responses.

### Model equations

The results from experiments using the Box–Behnken design were used to estimate the model coefficients. The fitted models expressed in coded variables and actual variables are represented by Eq. (2). The estimated model, expressed in coded variables, was:2

The estimated model, expressed in actual variables, was:  = 94.90 + 0.14 × pH + 0.73 × temperature − 50.38 × laccase + 8.09 × HBT + 0.02 × dye − 0.27 × time + 0.52 × pH × temperature + 15.65 × pH × laccase + 9.72 × pH × HBT − 0.08 × pH × dye + 0.13 × pH × time − 0.15 × temperature × laccase − 0.92 × temperature × HBT + 0.002 × temperature × dye − 0.004 × temperature × time − 11.67 × laccase × HBT + 0.08 × laccase × dye − 0.18 × laccase × time − 0.072 × HBT × dye − 0.082 × HBT × time + 0.001 × dye × time − 5.24 × pH^2^ − 0.025 × temperature^2^ + 3.24 × laccase^2^ + 11.49 × HBT^2^ + 0.0018 × dye^2^ − 7.44E−05 × time^2^.

The comparison of the decolourization yield of the actual value and the predicted value from the model showed that the run 42 was too different. For this reason, the model was adjusted after omitting results of experiment 42 (Eq. 2).

The estimated model, expressed in actual variables, was:  = 68.32 − 1.12 × pH + 1.06 × temperature − 24.49 × laccase + 29.23 × HBT + 0.07 × dye − 0.09 × time + 0.51 × pH × temperature + 2.97 × pH × laccase + 9.71 × pH × HBT − 0.08 × pH × dye + 0.04 × pH × time − 0.15 × temperature × laccase − 0.91 × temperature × HBT + 2.83 × temperature × dye − 4.37 × temperature × time − 11.67 × laccase × HBT + 0.08 × laccase × dye − 0.01 × laccase × time − 0.07 × HBT × dye − 0.08 × HBT × time + 1.16 × dye × time − 3.35 × pH^2^ − 0.02 × temperature^2^ + 13.8 × laccase^2^ − 9.64 × HBT^2^ + 1.34E−03 × dye^2^ + 3.95E–04 × time^2^.

### Analysis of variance and validation of the model

The statistical significance of the polynomial model for the experimental responses was calculated using analysis of variance (ANOVA). Table 4 shows that the regression sum of squares was statistically significant when using the *F* test at a 95% probability level, suggesting that the variation accounted for by the model was significantly greater than the unexplained variation. In addition, the seven check-point results were used to validate the fitted model (Table 5). The measured values *y*_*i*_ were very close to those calculated () using the model equation (Table 6). Moreover, the differences between calculated and measured responses were not significant (*t* test, *P* > 0.05). It was concluded, therefore, that the model determined for this dye was adequate in describing the response surfaces, and could be used as a prediction equation in the design space.

**Table 4:** Analysis of variance for response surface quadratic model of the Sirius Rose BB dye

Source of variation	Sum of squares	Degrees of freedom	Mean square	*F* value	*p* value Prob > *F*	Significance
Regression	5098.89	27	188.8	2.78	0.0061	*
Residuals	1699.78	25	67.99			
Cor total	6798.67	52				
*R* squared	0.750					

* indicates significant at *P* = 95%

**Table 5:** Experimental condition of the check-points

Run	X_1_	X_2_	X_3_	X_4_	X_5_	X_6_
1	3.0	60	0.1	0.9	46.7	70.0
2	4.0	40	0.1	0.1	20.0	30.0
3	4.0	30	0.4	0.9	100.0	150.0
4	6.0	40	0.1	0.4	100.0	150.0
5	4.9	55	0.5	0.5	98.8	144.5
6	5.3	37	0.7	0.9	98.5	150.0
7	5.2	42	0.6	0.9	97.5	149.1

**Table 6:** Validation of the model with the check-points

Run	*Y*_exp._	*Y*_calc._	Residual	|t.exp.|	Leverage (dU)	Significance (%)
	*y*_*i*_		*e* = *y*_*i*_ −			
1	57.8	67.0	−9.2	0.896	0.5324	37.9
2	92.2	87.5	4.7	0.465	0.4941	64.6
3	85.3	101.3	−16.0	1.617	0.4427	11.9
4	76.6	84.6	−8.0	0.788	0.5158	43.8
5	96.3	96.6	−0.3	0.034	0.2408	97.3
6	94.4	94.1	0.3	0.032	0.3071	97.5
7	91.3	95.8	−4.5	0.489	0.2354	62.9

### Graphical interpretation of the response surface model

**Fig. 2:**
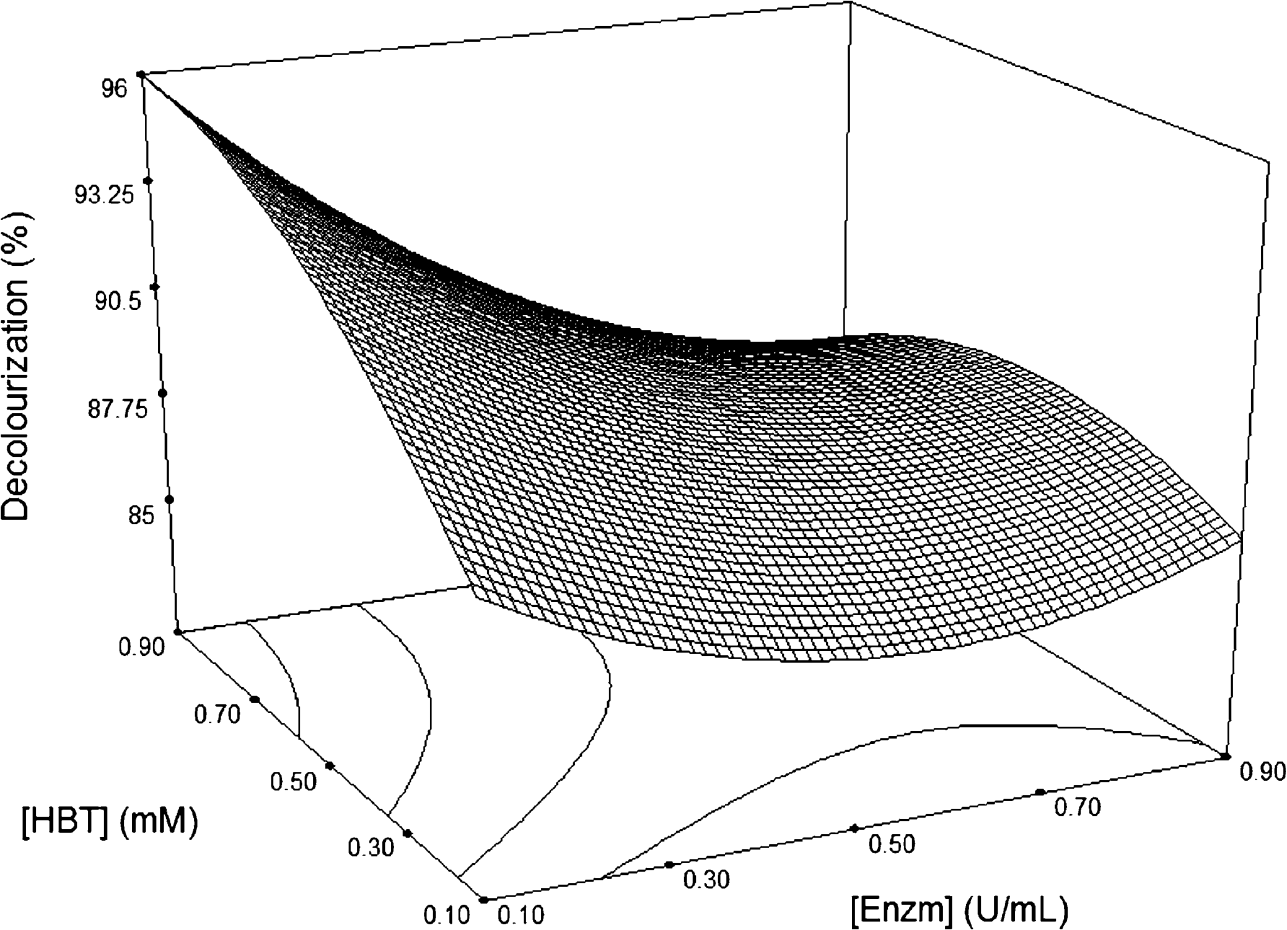
3D surface plot for the removal of Sirius rose (SR) by laccase as a function of 1 hydroxybenzotriazole (HBT) and enzyme concentrations, at a fixed value of 60 mg/L SR

**Fig. 3:**
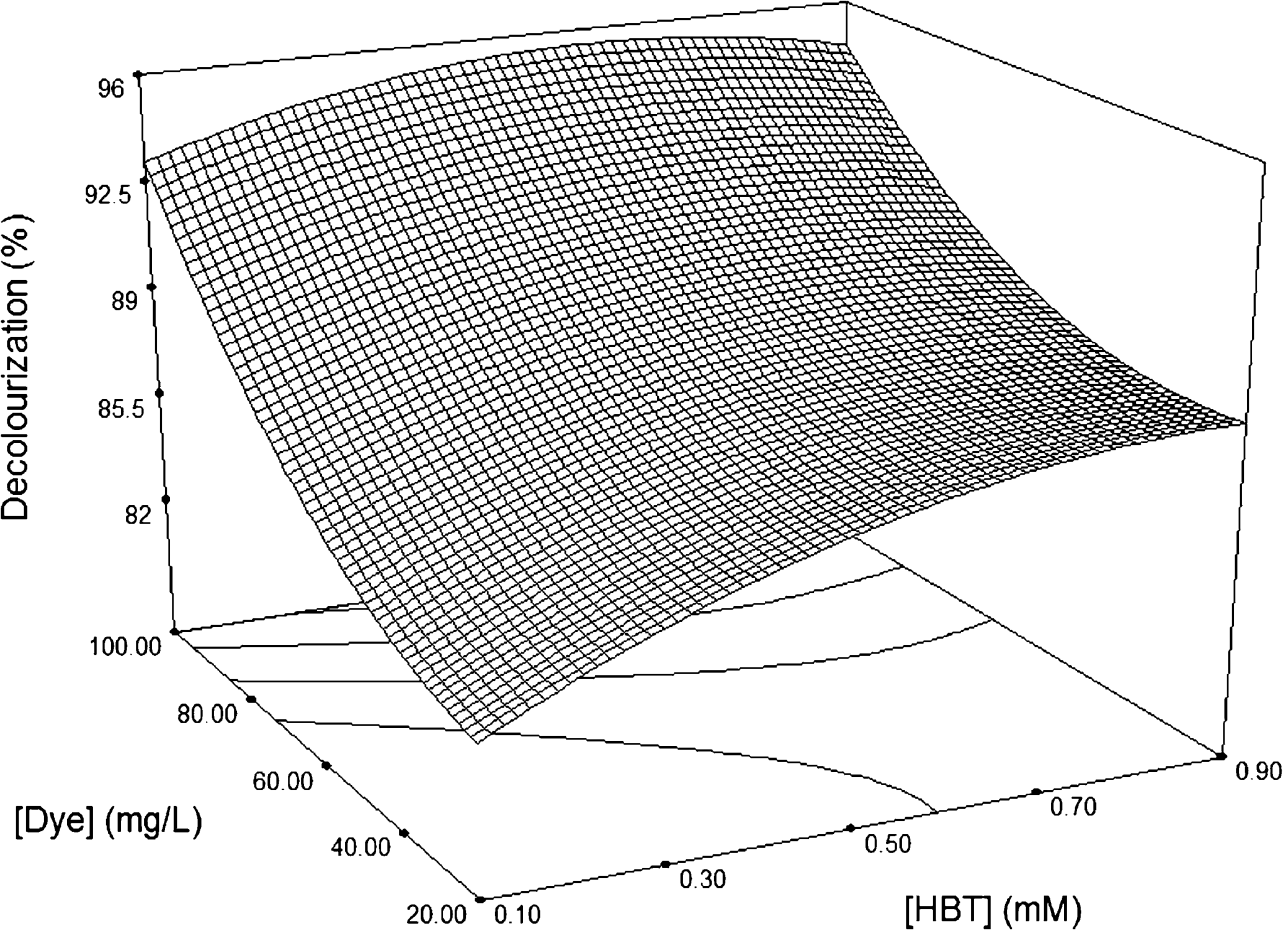
3D surface plot for the removal of Sirius rose (SR) by laccase as a function of 1 hydroxybenzotriazole (HBT) and dye concentrations, at a fixed value of 0.5 U/mL laccase

**Fig. 4:**
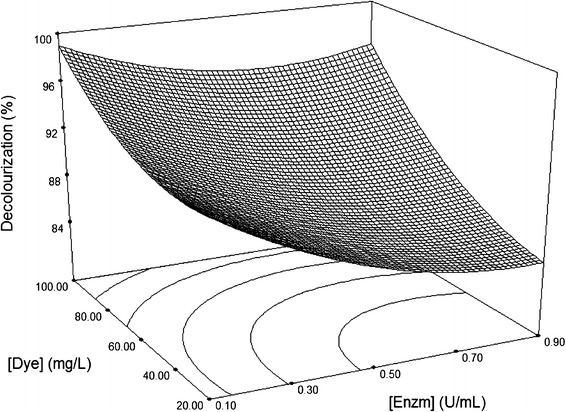
3D surface plot for the removal of Sirius rose (SR) by laccase as a function of dye and enzyme concentrations, at a fixed value of 0.5 mM HBT

**Fig. 5:**
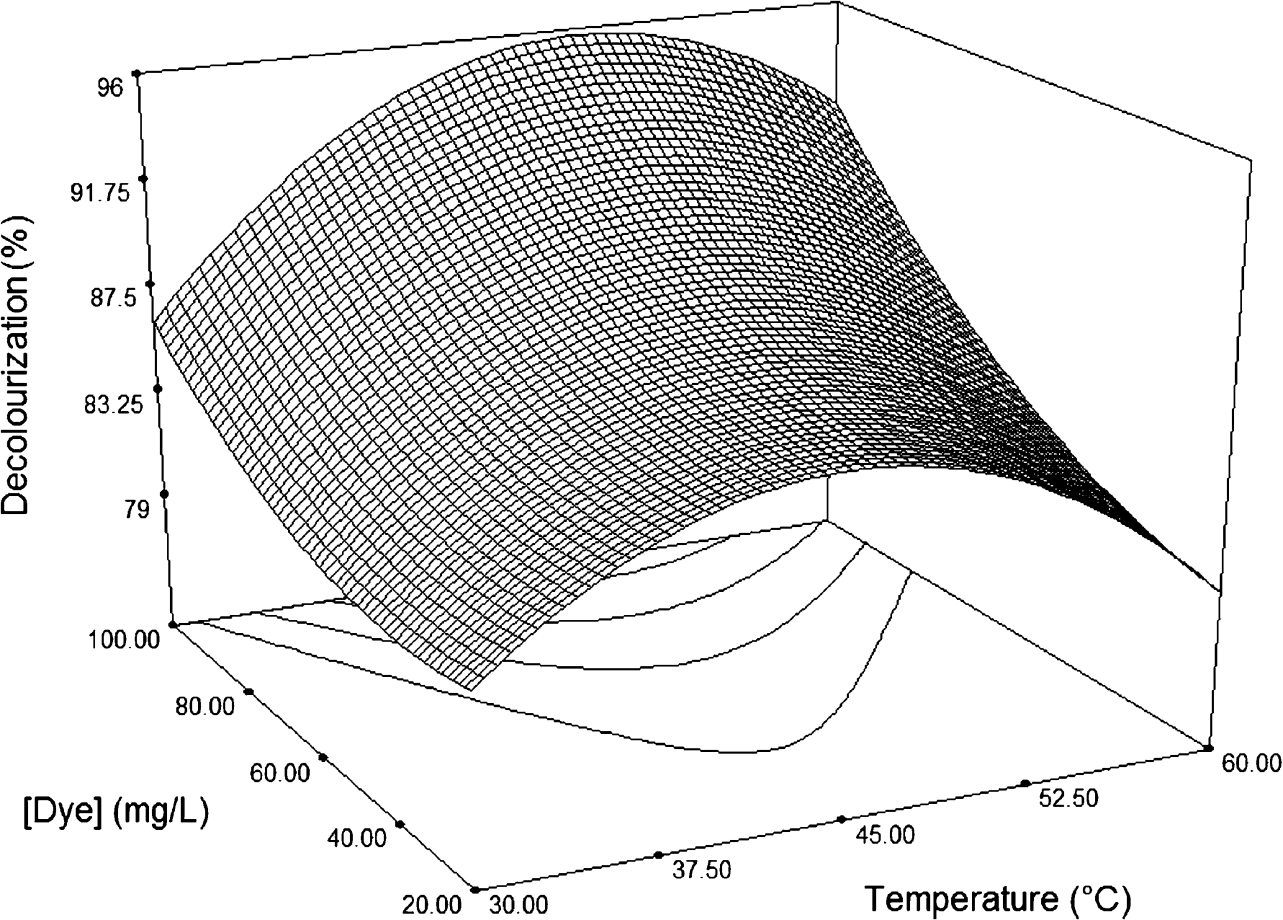
3D surface plot for the removal of Sirius rose (SR) by laccase as a function of dye concentrations and pH, at fixed values of 0.5 mM HBT, 0.5 U/mL laccase and 45 °C

**Fig. 6:**
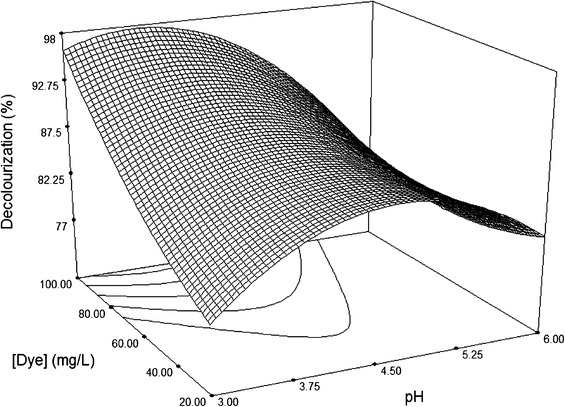
3D surface plot for the removal of Sirius rose (SR) by laccase as a function of dye concentrations and temperature, at fixed values of 0.5 mM HBT, 0.5 U/mL laccase and pH 4.5

Following validation of the model, the response surface and the isoresponse curves were prepared by plotting the response variation against two of variable factors, whilst a third factor was held constant at its mean level (0.5 U/mL of laccase, 0.5 mM HBT and 60 mg/L dye, pH 4.5 and 45 °C). The effects of these variables on decolouration of Sirius rose are shown in Figs. 2, 3, 4, 5,6.

The effect of HBT and enzyme concentrations on decolourization at a fixed dye concentration of 60 mg/L is shown in Fig. 2. Increasing concentrations of either HBT or laccase whilst maintain a fixed low value for the second factor resulted in an increase in the response surface. The response value reached its highest point at 0.9 mM HBT and 0.1 U/mL laccase. Increasing HBT and laccase concentrations over these values did not increase SR decolouration. In general, in enzymatic reactions the reaction rate increases with increasing substrate concentration. At a certain high value, the reaction rate reaches a plateau and is constant regardless of additional increases in substrate concentration (Cristóvão et al. 2008).

The effect of HBT and dye concentrations on the decolourization at a fixed laccase concentration of 0.5 U/mL is illustrated in Fig. 3. Increasing dye concentration appeared to result in an increase in the decolouration yield, irrespective of HBT concentration. This result differs from has previously been published on the effect of HBT on laccase activity. As a useful and important mediator of laccase activity in these reactions, the rate of decolourization generally increases with increasing HBT concentration (Kumarasamy et al. 2007). In this study, however, increases in HBT concentration at high dye concentrations had no notable impact on decolourization activity. Maximum dye decolouration was attained at HBT concentrations between 0.1 and 0.9 mM and 100 mg/L dye. In this context, and for a dye from the same family as Sirius rose, Zille et al. (2003) showed that the decolourization of Reactive Black 5 (RB5) was achieved with laccase of *Trametes villosa* without addition of a mediator. The redox potential of fungal laccase has been shown to vary depending on the source of laccase (Li et al. 1999). Other studies showed that HBT was essential for decolourization of remazol brilliant blue R (RBBR) by laccase from *Aspergillus*, but not for laccase from *Pycnoporus cinnabarinus* (Schliephake et al. 2000).

Figure 4 shows the effect of enzyme and dye concentrations at a fixed HBT concentration of 0.5 mM. Sirius rose decolouration increased with increase in dye concentration, regardless of the laccase concentration. Maximum decolourization was obtained at laccase concentrations of between 0.1 and 0.9 U/mL and 100 mg/L dye. Decolouration decreased with increased dye concentrations, even in the presence of high laccase concentrations. Similar effects have been reported previously with decolouration of RB5 by crude laccase from *Trametes pubescens* (Roriz et al. 2009).

The effects of temperature and pH at fixed HBT and enzyme concentrations of 0.5 mM and 0.5 U/mL, respectively, are shown in Figs. 5 and 6. These two plots had similar shapes and revealed that maximum of decolouration occurred at temperatures between 37 and 50 °C and pH values between 3.75 and 5, regardless of dye concentration. This range of temperature and pH is optimal for laccase activity, as proved in previous studies (Fortina et al. 1996).

Compared to others studies on the optimization of laccase–HBT system decolourization of azo dyes (Claus et al. 2002; Nyanhongo et al. 2002; Camarero et al. 2005; Murugesan et al. 2007; Kokol et al. 2006), the mathematical model presented in this study predicted the highest decolourization yield (99.5%) in shorter reaction time (150 min).

## Conclusion

Crude laccase enzyme was used as a biocatalyst for the decolouration of the diazo dye Sirius rose BB. RSM was successfully applied to determine the optimal operational conditions for maximum decolouration, which were found to be 99.5% at 45 °C, 60 mg/L dye, 0.5 mM HBT, 0.1 U/mL of laccase, citrate buffer pH 3, with 150 min incubation. A quadratic model, developed using these six factors, to represent decolouration percentage and the corresponding coefficients of independent variables, was estimated by the application of Design Expert 7.1 (trial version) and was highly significant.
